# Mycotic Pseudoaneurysm Associated with Skull Base Osteomyelitis Treated with Endovascular Embolization

**DOI:** 10.7759/cureus.1622

**Published:** 2017-08-28

**Authors:** Ali S Haider, Brandon I Esianor, Mrigank S Shail, Margaret I Engelhardt, Aida Kafai Golahmadi, Ramiz Khan, Umair Khan, Steven Vayalumkal, Richa Thakur, Kennith F Layton

**Affiliations:** 1 Neurosurgery, Texas A&M College of Medicine; 2 Otolaryngology, Mcgovern Medical School; 3 School of Medicine, Xavier University School of Medicine; 4 Neurosurgery, Imperial College London & Imperial College NHS Trust; 5 Bioengineering, UT Dallas; 6 School of Medicine, St. George's University; 7 Texas A&M College of Medicine; 8 Department of Radiology, Baylor University Medical Center

**Keywords:** : middle meningeal artery, mycotc pseudoaneurysm, otitis externa, osteomyelitis

## Abstract

Pseudoaneurysms occur due to malformations in arterial wall uniformity, leading to blood collection between the outer arterial layers and resultant outpouching of the vessel. Unlike true aneurysms, pseudoaneurysms do not involve all layers of the blood vessel. Mycotic pseudoaneurysms can occur after associated vessel adventitia infection, leading to transmural dissection. Here we present a case of a 78-year-old man with a history of chronic otitis externa and osteomyelitis who presented with increasing right ear pain with bloody discharge and associated headache. Catheter angiography demonstrated a large pseudoaneurysm in the right middle meningeal artery (MMA) at the base of the skull. Based on the clinical findings and the patient’s history, the patient was ultimately diagnosed with mycotic pseudoaneurysms of the MMA. The patient was subsequently treated with antibiotics as well as endovascular embolization and recovered without any complications.

## Introduction

Nontraumatic pseudoaneurysms of the middle meningeal artery (MMA) are rare, with approximately 27 cases having been reported as of March 2016 [1]. Formation of these pseudoaneurysms has been associated with various states of increased hemodynamic stress, such as Paget’s disease, meningiomas, arteriovenous malformations, Moyamoya disease, neurofibromatosis type II, cavernous hemangioma of the skull, and post aneurysm coiling [1]. However, pseudoaneurysms of the MMA associated with local spread of infection have yet to be reported in the medical literature. Mycotic pseudoaneurysms can occur after contiguous extension of an adjacent infection to the blood vessel adventitia with subsequent transmural dissection. This can lead to the formation of a false lumen, walled off by surrounding connective tissue [2]. Here, we present a unique case of MMA mycotic pseudoaneurysm as a complication of malignant otitis externa and chronic osteomyelitis, which was successfully treated with antibiotics and embolization.

## Case presentation

**Figure 1:**
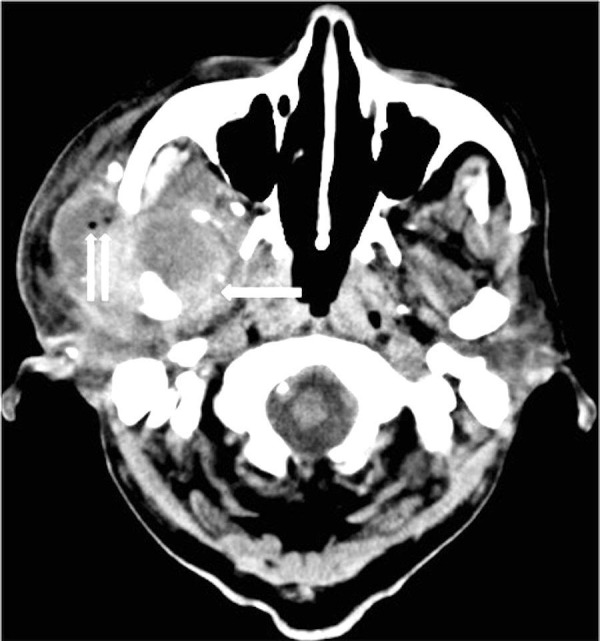
Noncontrast head computed tomography demonstrates heterogeneous mass in the right masticator space (arrow). Note the locules of air just lateral to the zygomatic arch (double arrow).

**Figure 2:**
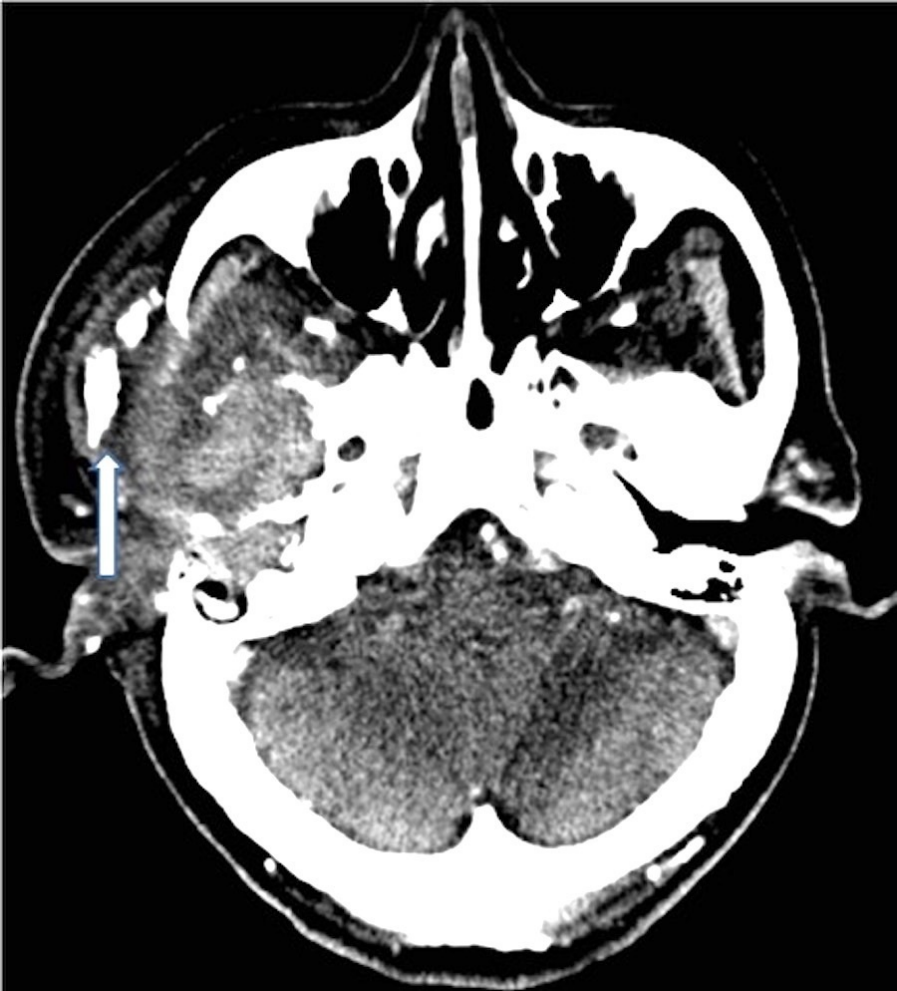
Contrast-enhanced computed tomography of the neck demonstrates the heterogeneous mass involving the right infratemporal fossa. There is contiguous destruction of the petrous temporal bone consistent with osteomyelitis. Note the bulky dystrophic calcifications lateral to the zygomatic arch (arrow).

A 78-year-old man with a history of end stage renal disease on hemodialysis, type II diabetes mellitus, and hypertension presented to an outside emergency department six days after the onset of increasing right ear pain with bloody discharge and associated headache. His vital signs were stable and neurological examination was unremarkable. The initial outside head and neck computerized tomography (CT) scans demonstrated what appeared to be a 2.6 cm mass in the infratemporal fossa and right parotid gland with destruction of the middle cranial fossa and right mandibular condyle (Figures 1-2).

**Figure 3:**
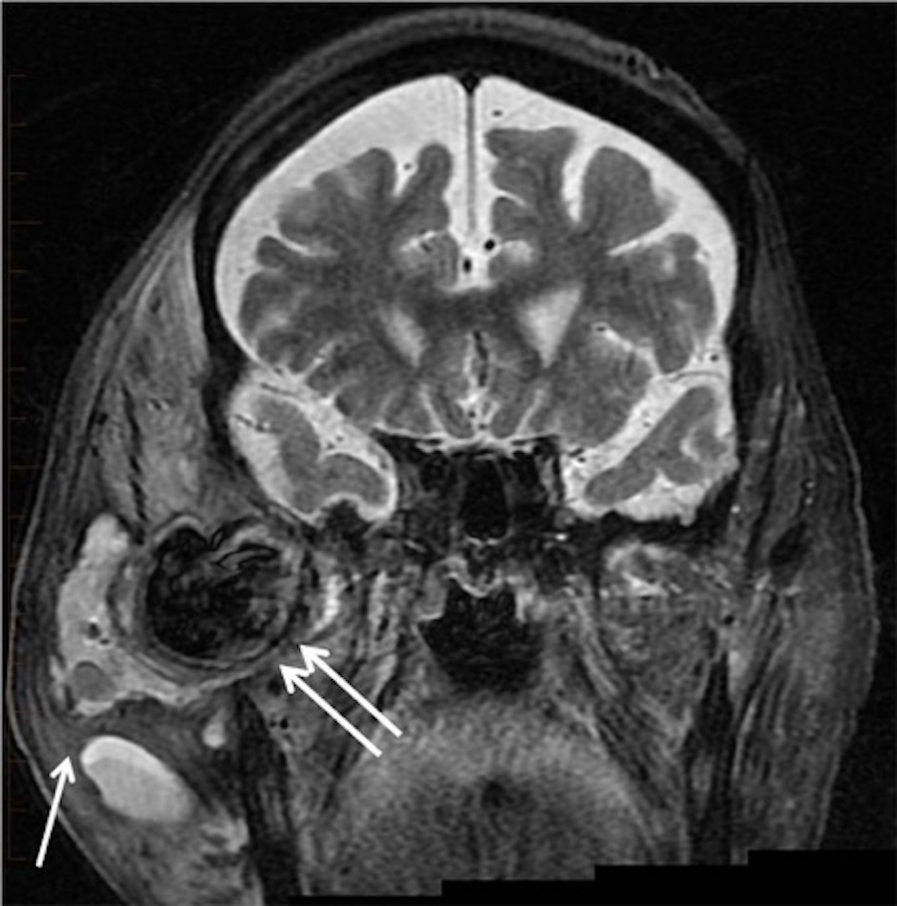
Coronal short tau inversion recovery magnetic resonance imaging reveals a multiloculated, hyperintense fluid collection in the soft tissue lateral to the mandible and zygomatic arch (arrow). Note the separate hypointense collection in the masticator space anterior to the mandibular condyle (double arrow). This is turbulent flow within the pseudoaneurysm.

Initial biopsy at an outside institution of the "destructive parotid mass" was unrevealing for neoplasm. He was subsequently transferred to Baylor University Medical Center for a higher level of care. Additional magnetic resonance imaging (MRI) of the skull base revealed a trans-spatial inflammatory process involving the right skull base and mastoids (Figure 3).

**Figure 4:**
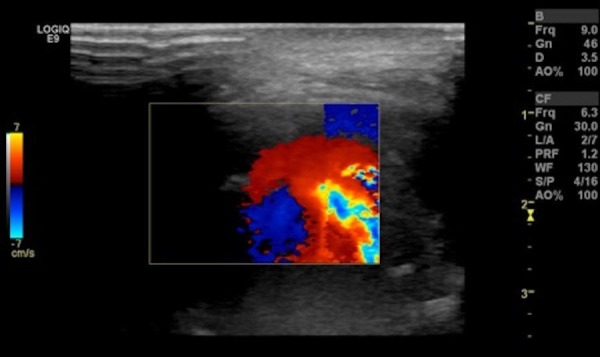
Ultrasound image of the right parotid region with Doppler interrogation reveals swirling flow within the large pseudoaneurysm in the right masticator space.

Ultrasound revealed a multi-compartmental fluid collection in the right parotid and masticator spaces including an area with swirling flow consistent with a pseudoaneurysm (Figure 4).

**Figure 5:**
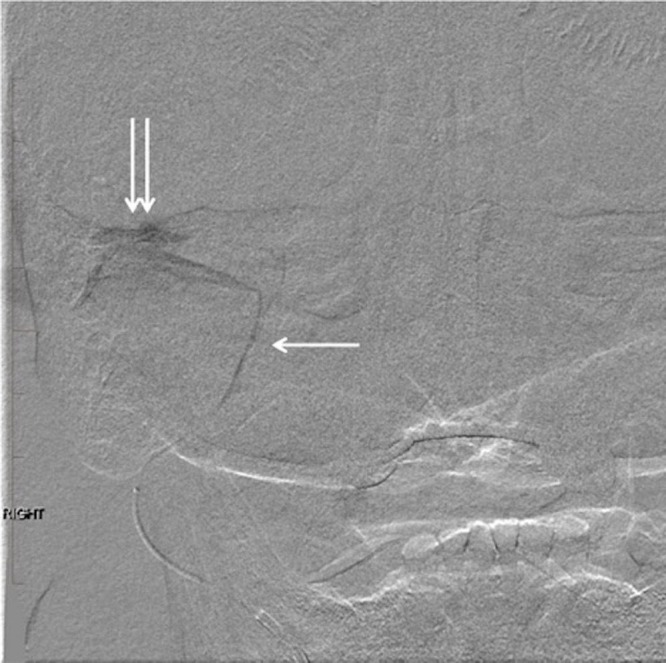
Digital subtraction angiogram with injection of the right middle meningeal artery. Note the microcatheter tip in the mid-segment of the right middle meningeal artery (arrow). There is a jet of contrast filling the large pseudoaneurysm in the soft tissues just beneath the right middle cranial fossa (double arrow). "Right" indicates the patient's right side.

**Figure 6:**
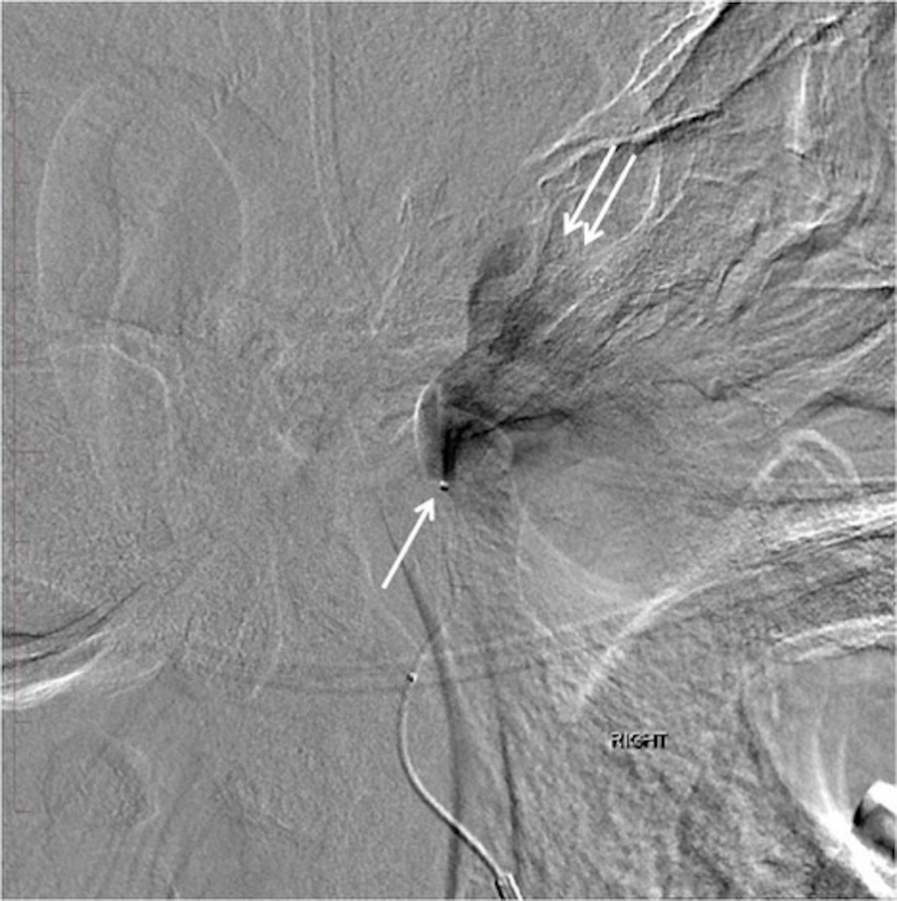
Lateral digital subtraction angiography showing the tip of the microcatheter in the mid-segment of the right middle meningeal artery (arrow). Note the jet of contrast filling the large pseudoaneurysm just below the base of the skull (double arrows). "Right" indicates the patient's right side.

Fine needle aspiration of a nonvascular fluid collection was performed. Catheter angiography demonstrated a large pseudoaneurysm involving the right MMA with contrast swirling into a large "mass" just below the skull base (Figures 5-6).

The etiology of the MMA pseudoaneurysm was postulated to be from the patient's chronic malignant otitis externa and chronic mastoid osteomyelitis. The causative organism was not isolated, as blood cultures and fluid aspiration showed no growth on follow-up.

**Figure 7:**
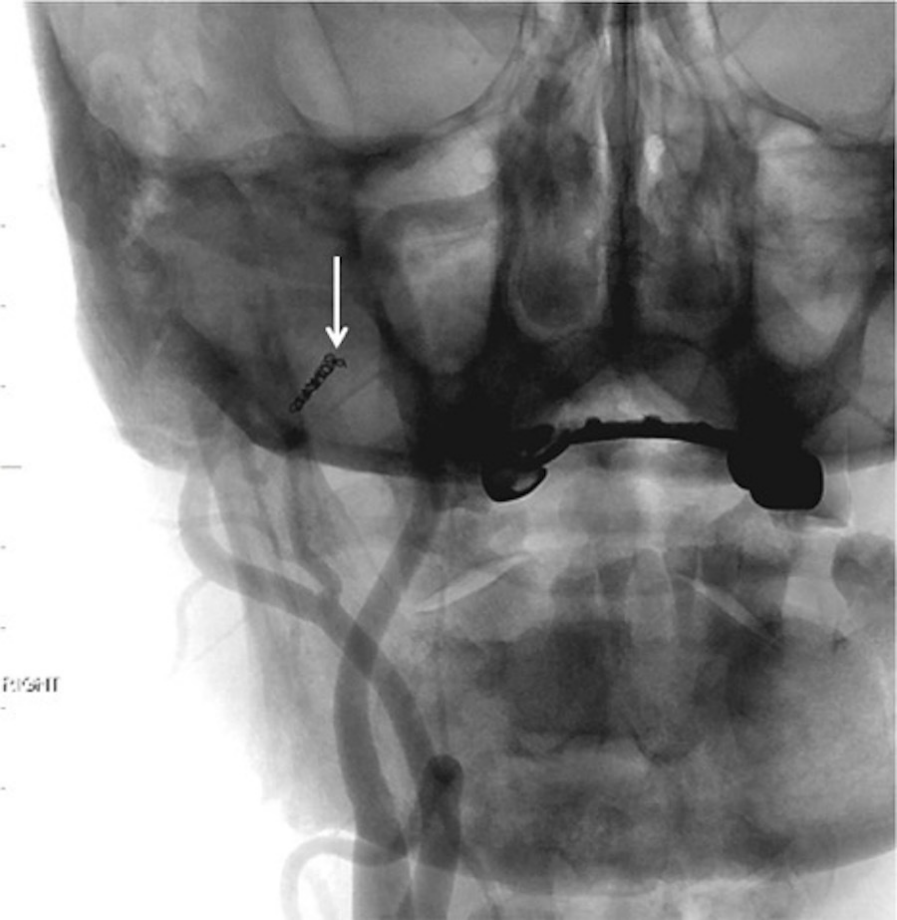
Native frontal projection fluoroscopic image following embolization of the right middle meningeal artery. Note the coil placed at the origin of the right middle meningeal artery (arrow). "Right" indicates the patient's right side.

To reduce the risk of significant bleed and resolve the ongoing bloody discharge and enlarging facial "mass", the patient underwent endovascular embolization of the extracranial right MMA pseudoaneurysm with the use of a custom-shaped Gelfoam torpedo at the fistula site and a single 2 mm platinum coil at the origin of the MMA (Figure 7).

**Figure 8:**
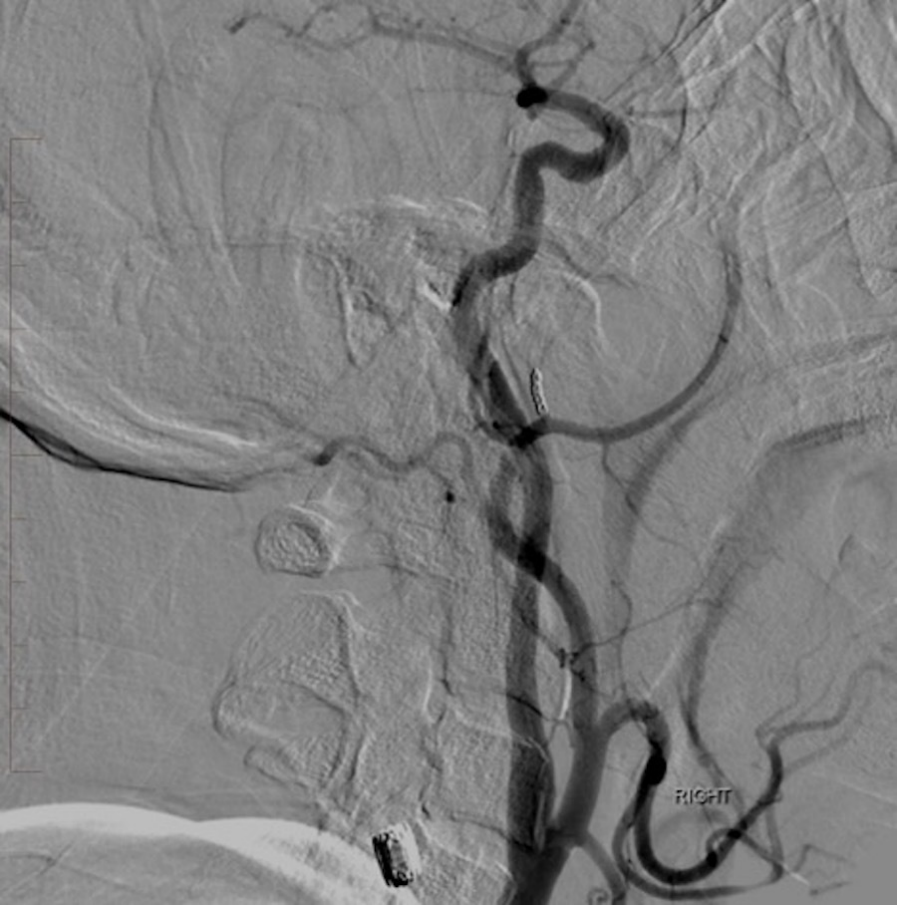
Lateral digital subtraction angiography of the right common carotid artery injection reveals complete occlusion of the pseudoaneurysm. The previously noted jet of contrast and large area of opacification are no longer visualized. "Right" indicates the patient's right side.

**Figure 9:**
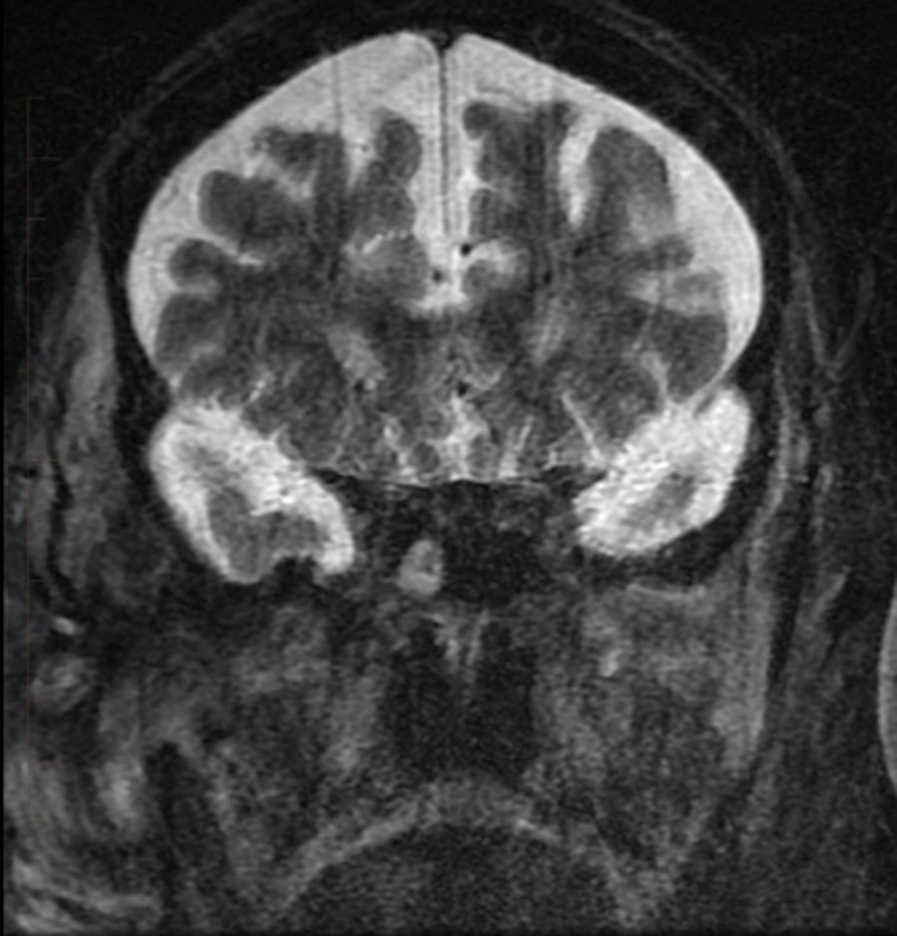
Follow-up coronal short tau inversion recovery magnetic resonance imaging five months after treatment demonstrating absence of the previously noted pseudoaneurysm and marked improvement of trans-spatial inflammatory changes.

Post-embolization angiography showed no filling of the pseudoaneurysm, indicating successful occlusion of the right MMA (Figures 8-9).

The patient tolerated the procedure well without any complications. To manage the infection, the patient was started on an eight-week course of intravenous piperacillin-tazobactam (Zosyn). He was discharged in afebrile condition, with a normal white blood cell count and decreased mass effect. Follow-up MRI of the skull base performed five months after discharged showed resolution of the pseudoaneurysm and improvement of inflammatory changes in the right parotid and masticator spaces.

## Discussion

Pseudoaneurysm of the MMA are rare, typically occurring secondary to trauma. As of March 2016, approximately 27 cases of non-traumatic pseudoaneurysm of the MMA have been reported in the medical literature [1]. No true guidelines currently exist regarding treatment. In previous cases, treatment had included craniotomy with ligation, resection, and endovascular techniques [1]. Positive results have been reported following both embolization and surgical resection of MMA pseudoaneurysm [3]. In our case reported here, an endovascular technique was used because the treatment team determined the patient to be a poor operative candidate. Furthermore, the large size of the pseudoaneurysm was considered prohibitive to safe open surgical ligation given the site of inflow to the pseudoaneurysm. The MMA pseudoaneurysm was embolized with Gelfoam at the fistula site and a single 2 mm coil at the MMA origin. Gelfoam causes a temporary mechanical obstruction, reduces blood flow, and stimulates thrombus formation. The use of coils for embolization causes permanent occlusion equivalent to that of surgical ligation [4]. Of note, colonization of coils by microorganisms can occur during coil insertion in the setting of bacteremia or local infection [5]. In two previous cases, the proximal MMA was embolized using n-butyl cyanoacrylate, a synthetic glue [6-7]. Balloon-assisted low viscosity Onyx embolization has been reported to be an effective method for emergency treatment of infected pseudoaneurysms while maintaining parent artery patency [8]. In our case, the coil was placed at a distance from the site of the mycotic pseudoaneurysm at the origin of the MMA in order to avoid an additional nidus for infectious complications.

The formation of this patient’s pseudoaneurysm was multifactorial in nature. He presented with a long-term history of type II diabetes mellitus, a possible risk factor for infected pseudoaneurysm formation due to the damaging effect on blood vessels [9]. Additionally, the patient presented with chronic malignant external otitis (MEO), a rare necrotizing infection of the external auditory canal and skull base often associated with Pseudomonas aeruginosa. Other bacterial agents that cause MEO include Staphylococcus aureus, Proteus mirabilis, Klebsiella oxytoca, and Pseudomonas cepacia [10]. In patients with type II diabetes mellitus, the risk of developing MEO increases proportionally to their age. In advanced-disease cases such as the one we present, skull base osteomyelitis can develop. The most effective treatment method of MEO is to ensure diabetes is well managed, control infection with systemic antibiotics, debride necrotic tissue, and surgically manage when necessary [10]. In this case, priority was given towards the treatment of the MMA pseudoaneurysm due to the high risk of rupture, which can be fatal. Monitoring of therapeutic response can be achieved by tracking normalization of erythrocyte sedimentation rate (ESR) as well as imaging surveillance as in this case [10].

## Conclusions

Pseudoaneurysms of the MMA are commonly associated with trauma; however, nontraumatic etiologies can also result in pseudoaneurysm formation. Here we presented the case of an infection-induced pseudoaneurysm which was subsequently treated with antibiotics and endovascular embolization. It is important to recognize nontraumatic causes of pseudoaneurysm formation so that prompt treatment can be initiated and fatal outcomes can be avoided.
